# Supplementary material for: Identifying regulators of aged fibroblast activation in 3D tissue models
Source: Sci Rep. 2025 Nov 26;15:42119. doi: 10.1038/s41598-025-25997-z (PMC12657520; doi:10.1038/s41598-025-25997-z)
Supplement: Supplementary file 1 — Supplementary Material 1 [file 41598_2025_25997_MOESM1_ESM.pdf]

Identifying regulators of aged fibroblast activation in 3D tissue models

Hui Liu[1,2], Luezhen Yuan[1,2,3], G.V. Shivashankar[1,2,*]

1. Division of Biology and Chemistry, Paul Scherrer Institut, 5232, Villigen, Switzerland

2. Department of Health Sciences and Technology, ETH Zurich, 8092, Zurich, Switzerland

3. Broad Institute of MIT & Harvard, Cambridge, MA, USA

* To whom correspondence should be addressed; E-mail: gshivasha@ethz.ch

Supplementary material

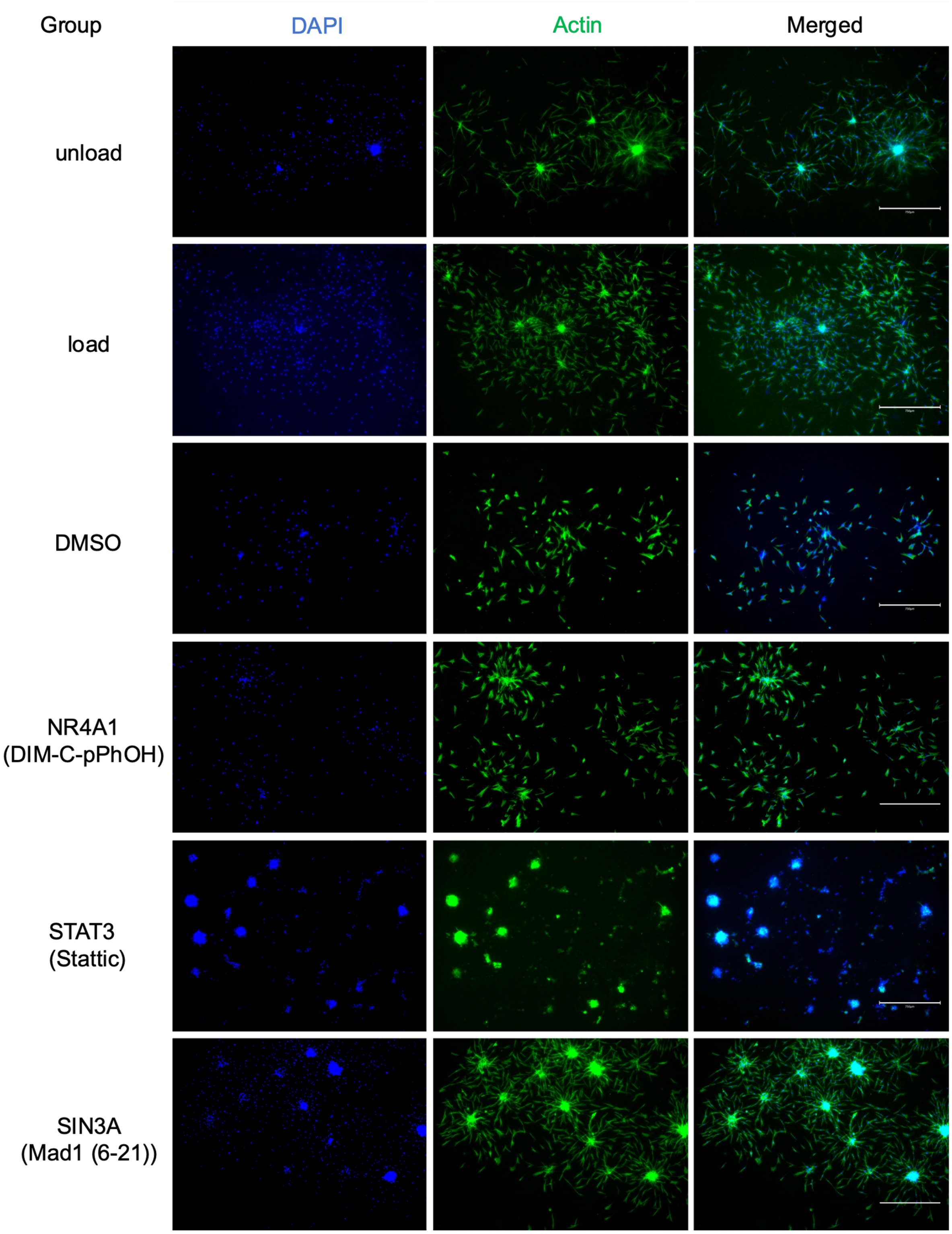
Group
DAPI
Actin
Merged
unload
load
DMSO
NR4A1
(DIM-C-pPhOH)
STAT3
(Stattic)
SIN3A
(Mad1 (6-21))

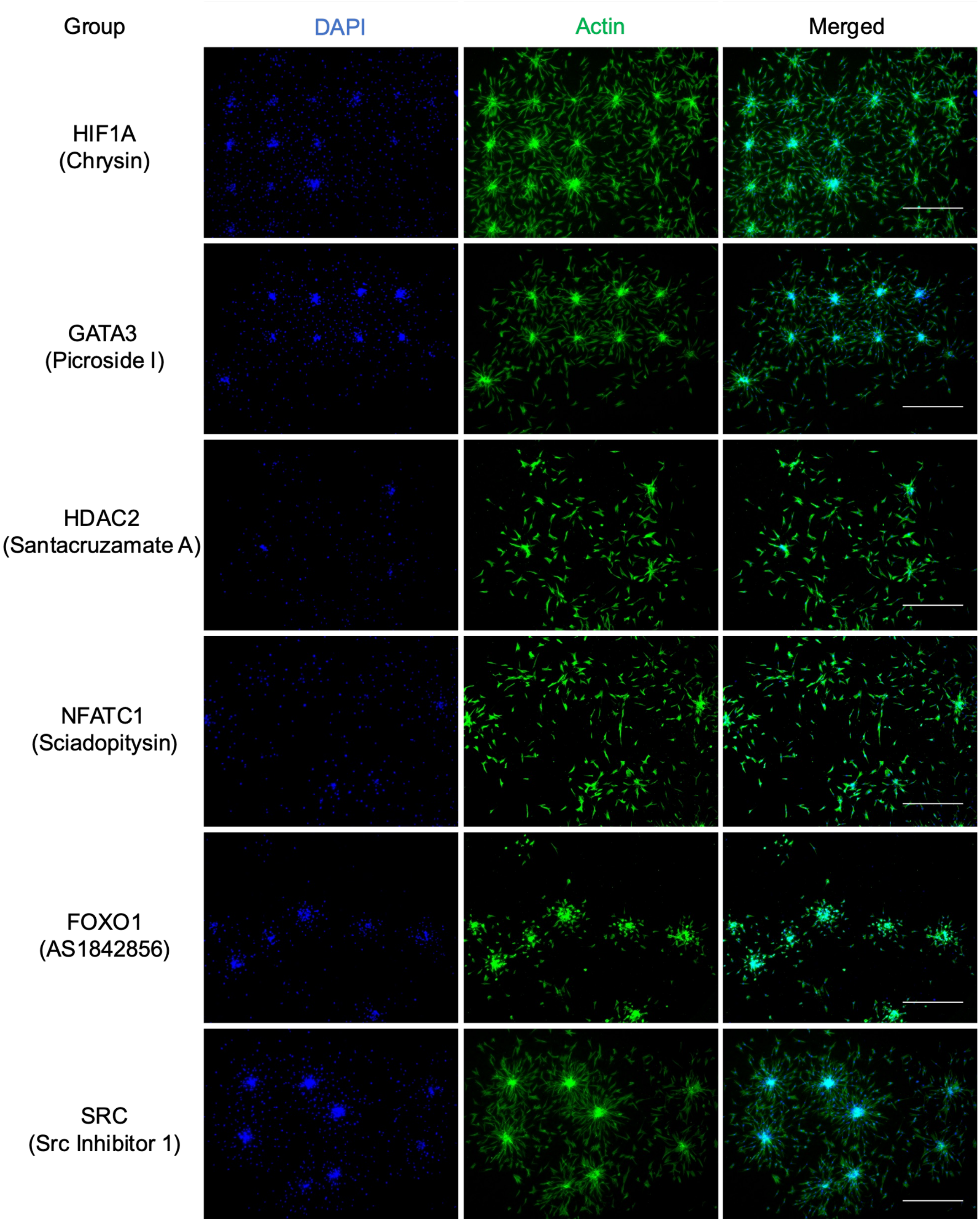
Group
DAPI
Actin
Merged
HIF1A (Chrysin)
GATA3 (Picroside I)
HDAC2 (Santacruzamate A)
NFATC1 (Sciadopitysin)
FOXO1 (AS1842856)
SRC (Src Inhibitor 1)

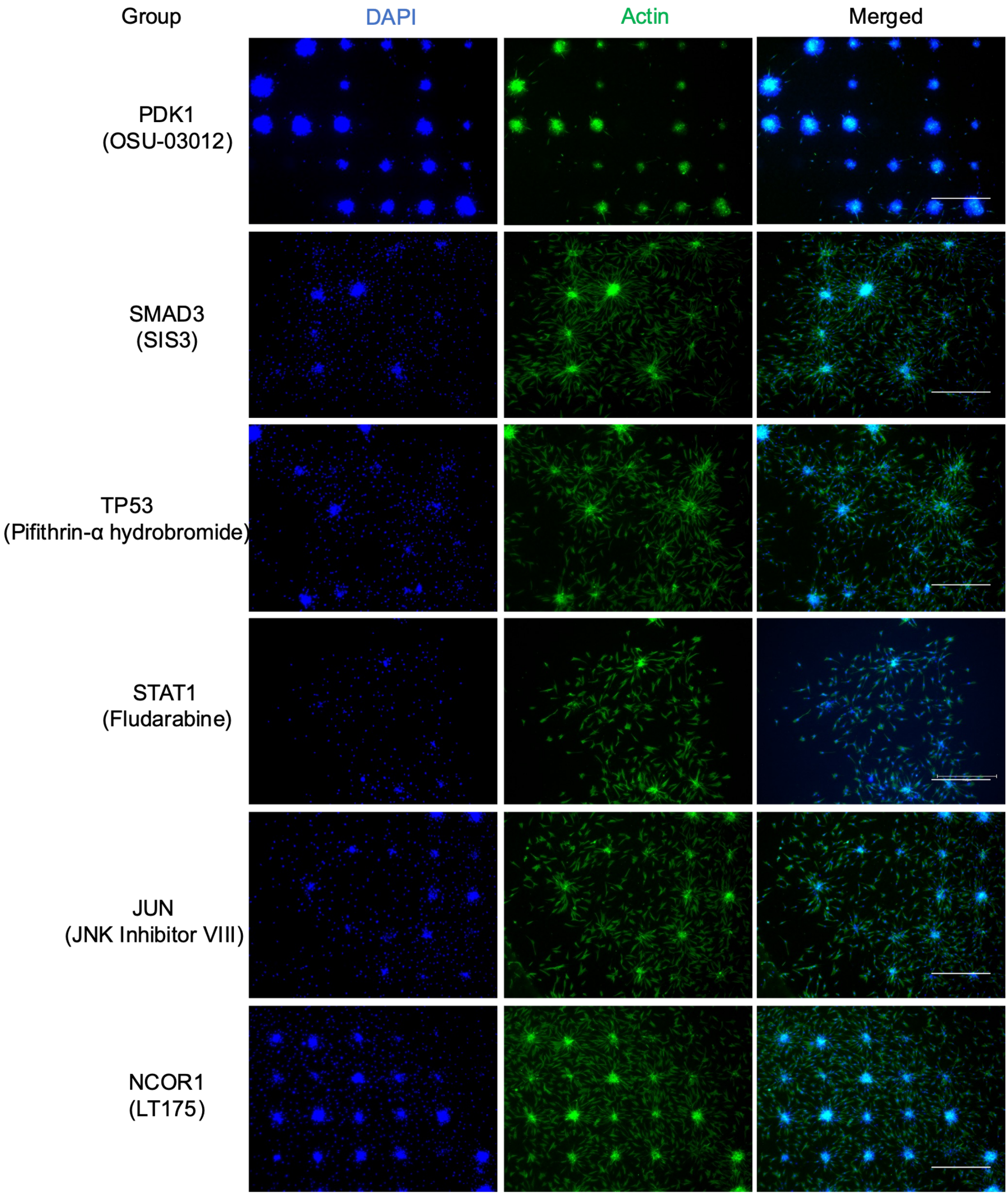

Figure S1. Big areas for TF drug screening (EVOS, 4X objective). (Scale bar, 750 μm). Green color (actin), Blue color (DAPI).

A

TF	Log2FoldChange
NR4A1	1.24948
CEBPD	1.39216
KLF9	1.07252
ZNF331	1.1721
IRF1	1.10464
NR6A1	1.28187

B

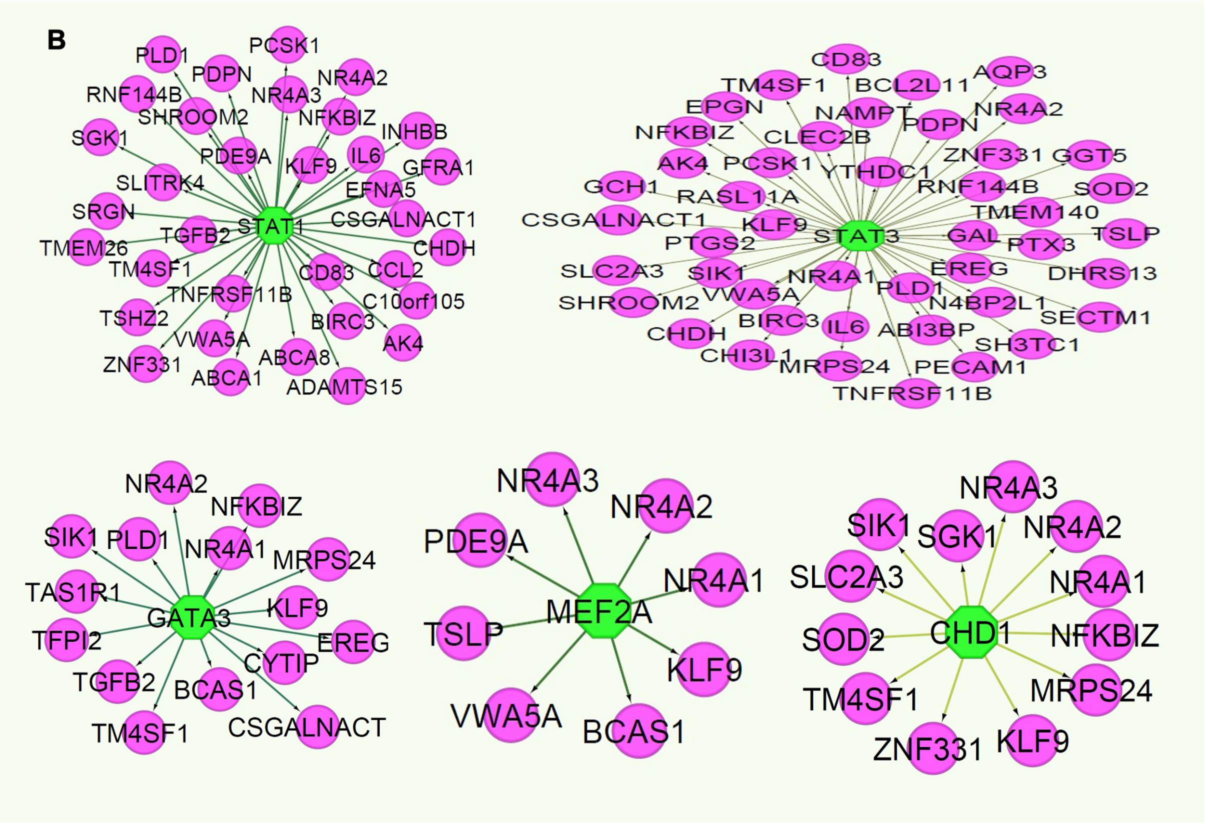

Figure S2. (A) Log2FoldChange of the most differentially expressed transcriptional regulators comparing 2× load and unload control group from TF nodes (in the pink color) in Figure 2A. (B) Regulatory network of representative TFs (STAT1, STAT3, GATA3, MEF2A and CHD1) and their target genes, pink nodes represent gene; green nodes represent TF.

A

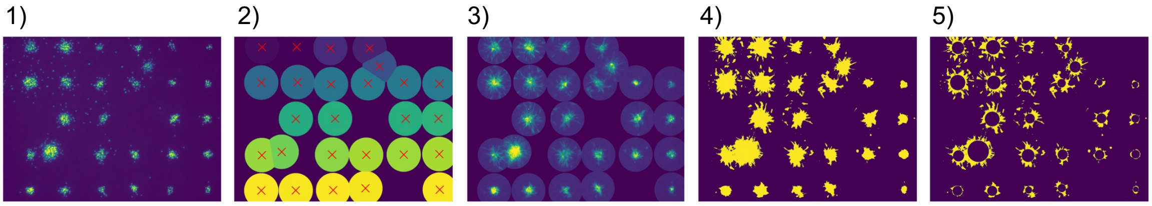

B

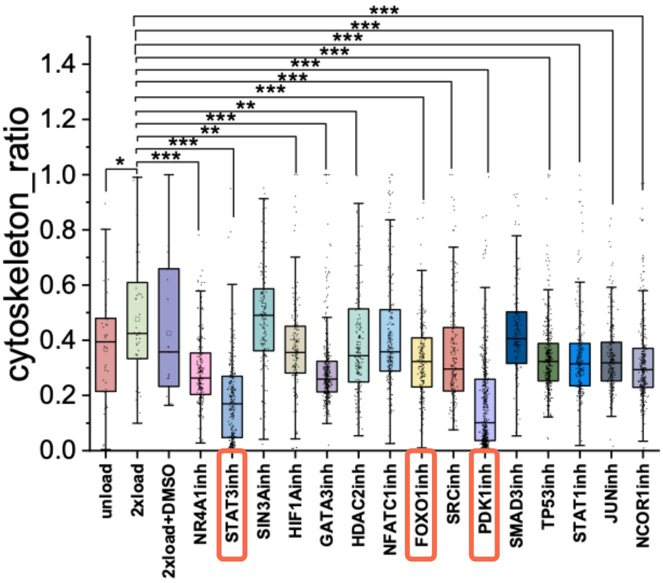

Figure S3. (A) Quantification pipeline for migration assay. DAPI images of spheroids were first segmented to estimate spheroid center. A ROI centered on an inferred spheroid center with diameter of 250 μm was segmented for downstream analysis. Actin stained images within ROI were segmented and the core of the spheroid were estimated using distance_transform_edt from Scipy. (B) Boxplot showing outside of the core of the spheroid but within ROI, the ratio of cytoskeleton area. Each group has about 20~550 single spheroids (as individual data points), from over 10 random field of views. P values were calculated by unpaired, two-tailed Student’s t test, compared to the 2×load group. *$P<0.05$; **<0.01; ***$P<0.001$.

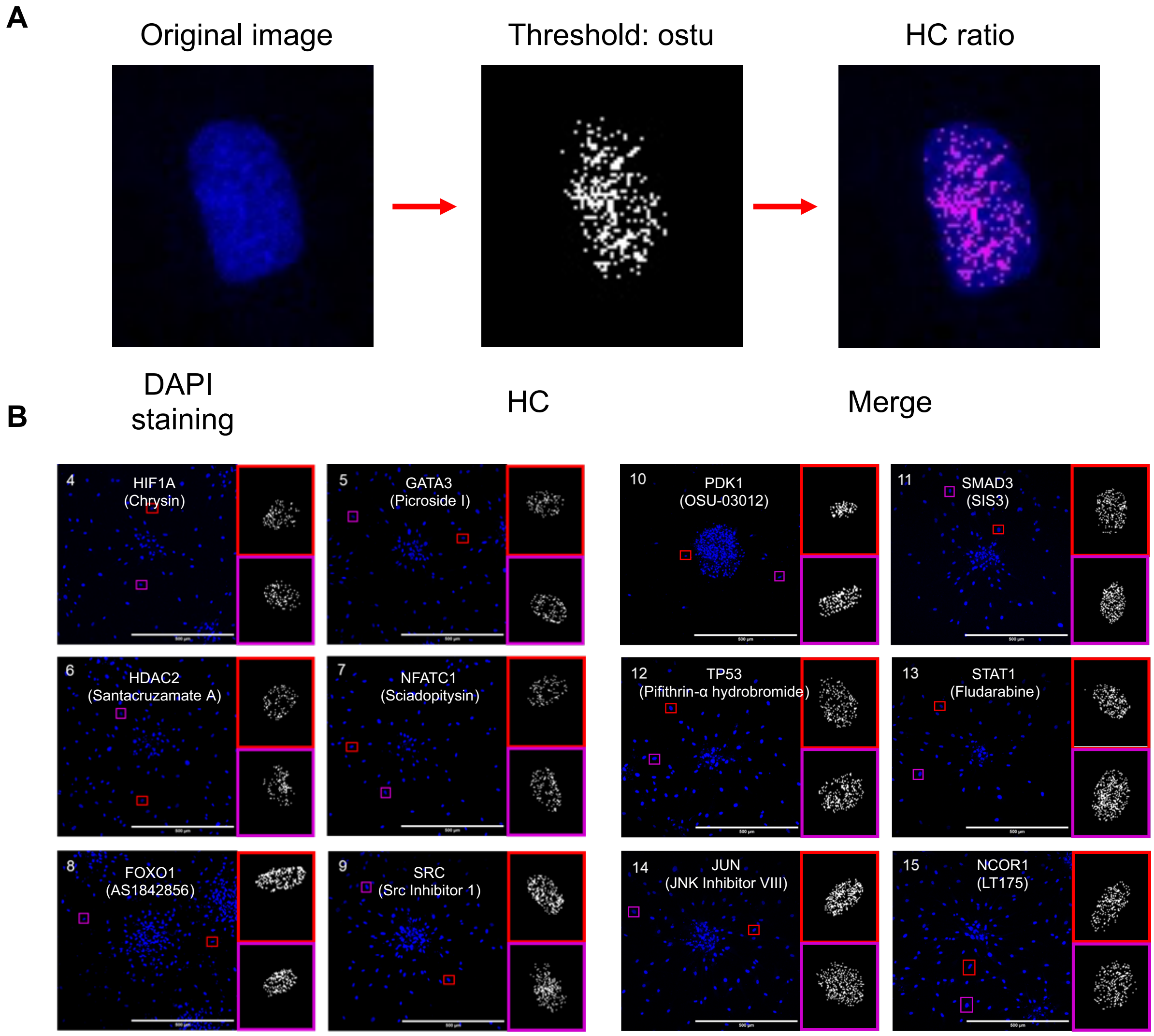

Figure S4. (A) Schematic of measuring the heterochromatin (HC). (B) Representative DAPI stained image showing heterochromatin distribution in cells from experiments in Figure3. Insert: Ostu thresholded dense chromatin regions were shown. Blue compounds: low level of i80_i20 compared to 2×load group. Red compounds: higher level than 2×load group.

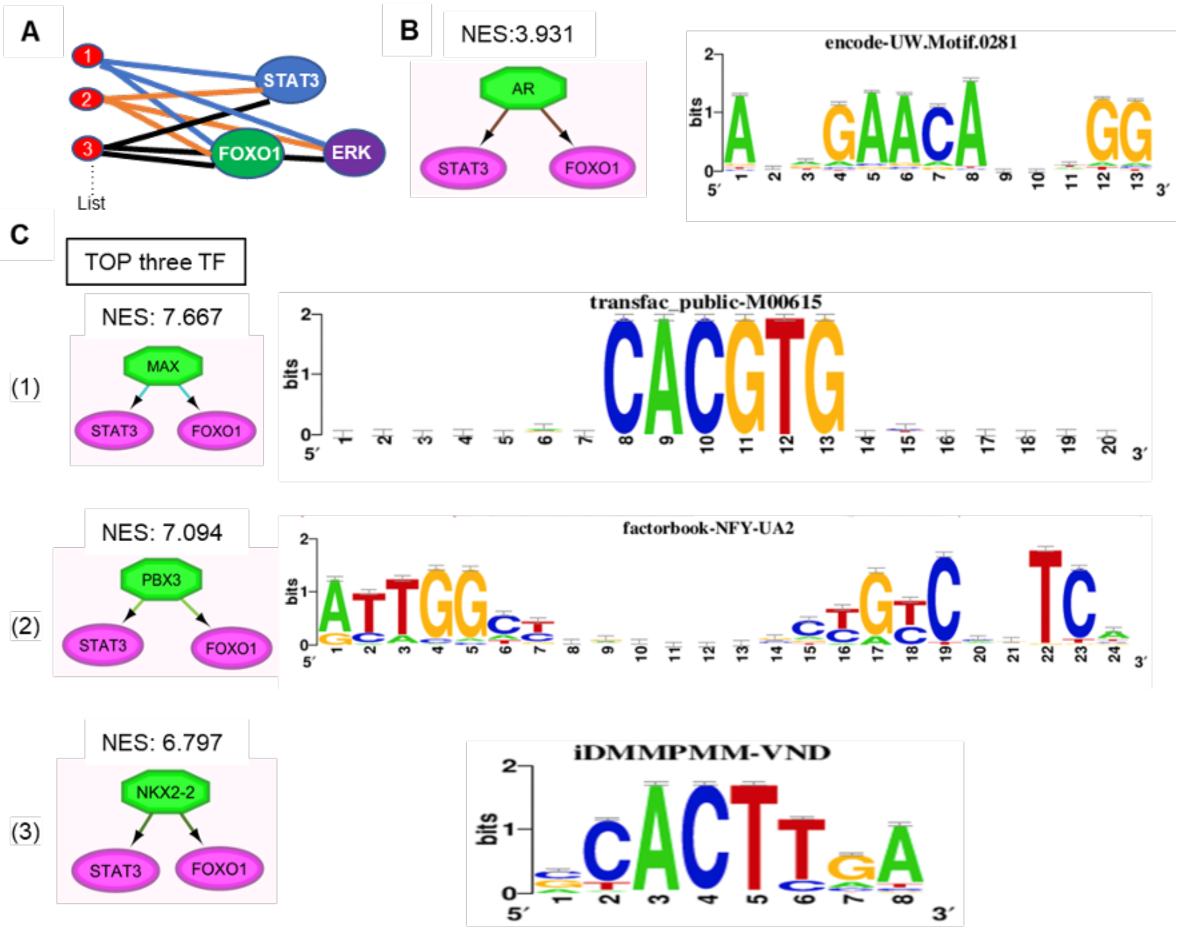

D Others:

TF name	XBP1	POU4F2	ONECUT1	PAX2	TBP	PAX7	NKX2-8	PAX5	NFYA
NES	6.514	6.457	6.358	6.153	5.841	5.516	5.509	5.466	5.275

TF name	STAT3	PRDM2	FOXP3	GATA2	GMEB1	NR1I2	EP300	MYCN	IRF7
NES	5.154	5.034	4.320	4.298	4.277	4.214	4.191	4.185	4.171

TF name	METTL3	NR6A1	RARA	C19orf40	PBX3	BATF	NFAT5	SIX1	OTX2
NES	4.157	4.143	4.072	4.008	3.996	3.985	3.980	3.945	3.923

TF name	ARID3A	KLF4	IRF6	JUND	PQBP1	STAT1	NFYA	MAX
NES	3.902	3.881	3.860	3.818	3.357	3.322	3.300	3.233

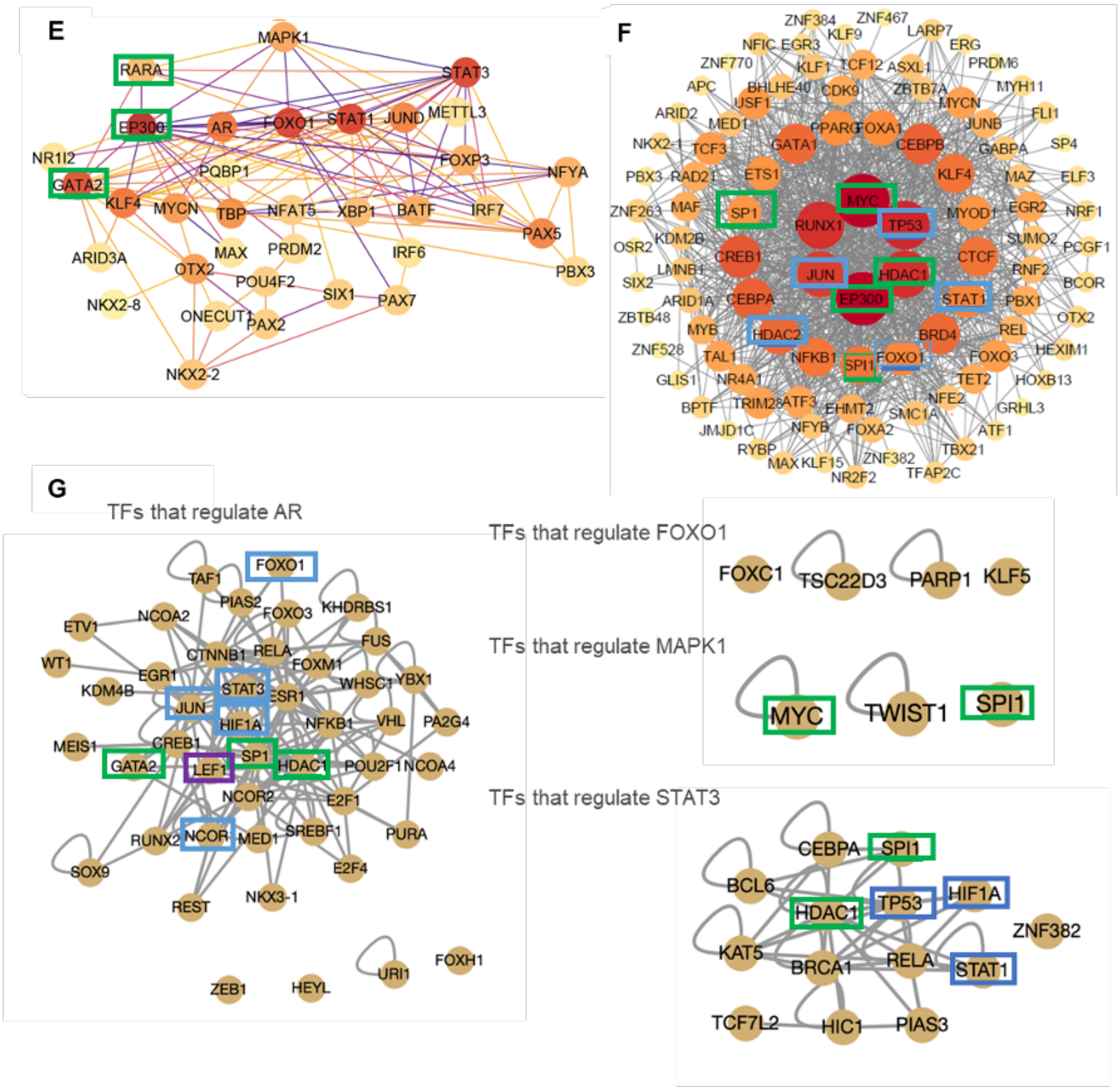

Figure S5. Potential upstream regulators of ERK, STAT3 and FOXO1. (A) Diagram for upstream list for ERK, STAT3 and FOXO1. (B-D) Prediction upstream regulators of STAT3 and FOXO1 by cytoscape plugin iRegulon. Representative motif plot for AR, MAX, PBX3 and NKX2-2. Table for other potential TF which regulate STAT3 and FOXO1 and their NES. (E) PPI network between ERK (MAPK1) and potential upstream regulators of STAT3 and FOXO1, which comes from (B-D). (F) PPI network of TF which regulated three ERK, STAT3 and FOXO1 simultaneously. These TF select from prediction function in database of human transcription factor targets (https://guolab.wchscu.cn/hTFtarget/#!/prediction). (G) PPI network of TF which regulated AR, ERK, STAT3 and FOXO1 separately. These TF select from prediction function in database of TRUST. Among E-G, Blue box, has been verified by drug screening (Figure 3.4); Green box, potential candidate; Purple box, has been studied in our lab.

Table S1. List of inhibitors. (* recommendation from supplier)

Drug name	Brand & catalogue number	Final concentration
DIM-C-pPhOH	MCE/HY-112055	15 uM (1)
Stattic	MCE/HY-13818	3.3 uM (2)
Mad1 (6-21)	MCE/HY-P3242	60nM
Chrysin	MCE/HY-14589	20 uM (3)
Picroside I	MCE/HY-N0407	10uM (4)
Santacruzamate A	MCE/HY-N0931	50uM
Sciadopitysin	MCE/HY-N2119	10uM (5)
AS1842856	MCE/HY-100596	5uM (6)
Src Inhibitor 1	MCE/HY-101053	10uM (7)
OSU-03012	MCE/HY-10547	20uM (8)
SIS3	MCE/HY-13013	10uM (9)
Pifithrin-α hydrobromide	MCE/HY-15484	10uM (10)
Fludarabine	MCE/HY-B0069	5uM (11)
JNK Inhibitor VIII	MCE/HY-107598	50nM (12)
LT175	MCE/HY-121900	5uM

Table S2. List of 119 differentially expressed genes used in TF identification.

Ensembl ID	Gene Name	Ensembl ID	Gene Name	Ensembl ID	Gene Name
ENSG00000165029	ABCA1	ENSG00000069482	GAL	ENSG00000073756	PTGS2
ENSG00000154262	ABCA6	ENSG00000131386	GALNT15	ENSG00000163661	PTX3
ENSG00000141338	ABCA8	ENSG00000131979	GCH1	ENSG00000122035	RASL11A
ENSG00000154258	ABCA9	ENSG00000151892	GFRA1	ENSG00000137393	RNF144B
ENSG00000204574	ABCF1	ENSG00000099998	GGT5	ENSG00000085721	RRN3
ENSG00000154175	ABI3BP	ENSG00000183474	GTF2H2C	ENSG00000141574	SECTM1
ENSG00000166106	ADAMTS15	ENSG00000019991	HGF	ENSG00000135919	SERPINE2
ENSG00000127507	ADGRE2	ENSG00000185885	IFITM1	ENSG00000118515	SGK1

ENSG00000162433	AK4	ENSG00000115598	IL1RL2	ENSG00000125089	SH3TC1
ENSG00000151150	ANK3	ENSG00000136244	IL6	ENSG00000146950	SHROOM2
ENSG00000165272	AQP3	ENSG00000163083	INHBB	ENSG00000142178	SIK1
ENSG00000109321	AREG	ENSG00000227811	INKA2-AS1	ENSG00000059804	SLC2A3
ENSG00000064787	BCAS1	ENSG00000119138	KLF9	ENSG00000179542	SLITRK4
ENSG00000153094	BCL2L11	ENSG00000291028	LINC00933	ENSG00000291237	SOD2
ENSG00000168398	BDKRB2	ENSG00000230537	LINC02937	ENSG00000122862	SRGN
ENSG00000023445	BIRC3	ENSG00000228536	LYPLAL1-AS1	ENSG00000173662	TAS1R1
ENSG00000214688	C10orf105	ENSG00000102802	MEDAG	ENSG00000105825	TFPI2
ENSG00000204536	CCHCR1	ENSG00000196611	MMP1	ENSG00000092969	TGFB2
ENSG00000108691	CCL2	ENSG00000166670	MMP10	ENSG00000169908	TM4SF1
ENSG00000108688	CCL7	ENSG00000137745	MMP13	ENSG00000146859	TMEM140
ENSG00000064205	CCN5	ENSG00000062582	MRPS24	ENSG00000196932	TMEM26
ENSG00000112149	CD83	ENSG00000139597	N4BP2L1	ENSG00000142188	TMEM50B
ENSG00000103888	CEMIP	ENSG00000105835	NAMPT	ENSG00000164761	TNFRSF11B
ENSG00000016391	CHDH	ENSG00000229644	NAMPTP1	ENSG00000181634	TNFSF15
ENSG00000133048	CHI3L1	ENSG00000277586	NEFL	ENSG00000182463	TSHZ2
ENSG00000133019	CHRM3	ENSG00000144802	NFKBIZ	ENSG00000145777	TSLP
ENSG00000110852	CLEC2B	ENSG00000171246	NPTX1	ENSG00000148154	UGCG
ENSG00000158270	COLEC12	ENSG00000123358	NR4A1	ENSG00000110002	VWA5A
ENSG00000147408	CSGALNACT1	ENSG00000153234	NR4A2	ENSG00000085741	WNT11
ENSG00000124875	CXCL6	ENSG00000119508	NR4A3	ENSG00000108379	WNT3
ENSG00000115165	CYTIP	ENSG00000065320	NTN1	ENSG00000083896	YTHDC1
ENSG00000121690	DEPDC7	ENSG00000175426	PCSK1	ENSG00000130844	ZNF331
ENSG00000167536	DHRS13	ENSG00000152270	PDE3B	ENSG00000227482	
ENSG00000115380	EFEMP1	ENSG00000160191	PDE9A	ENSG00000236453	
ENSG00000184349	EFNA5	ENSG00000004799	PDK4	ENSG00000237422	
ENSG00000138792	ENPEP	ENSG00000162493	PDPN	ENSG00000275993	
ENSG00000182585	EPGN	ENSG00000261371	PECAM1	ENSG00000283175	
ENSG00000124882	EREG	ENSG00000075651	PLD1	ENSG00000286694	
ENSG00000127533	F2RL3	ENSG00000240694	PNMA2	ENSG00000289322	
ENSG00000108950	FAM20A	ENSG00000159182	PRAC1		

1. Z. Ye, *et al.*, FBW7-NRA41-SCD1 axis synchronously regulates apoptosis and ferroptosis in pancreatic cancer cells. *Redox Biol.* **38**, 101807 (2021).

2. A. Bodac, *et al.*, Bcl-xL targeting eliminates ageing tumor-promoting neutrophils and inhibits lung tumor growth. *EMBO Mol. Med.* **16**, 158–184 (2024).

3. N. Li, Y. Liu, J.-R. Li, W.-X. Zhang, Chrysin, which targets PLAU, protects PC12 cells from OGD/R-stimulated damage through repressing the NF-κB signaling pathway. *Regen. Ther.* **19**, 69–76 (2022).

4. D. Rathee, M. Thanki, S. Bhuva, S. Anandjiwala, R. Agrawal, Iridoid glycosides-Kutkin, Picroside I, and Kutkoside from Picrorrhiza kurroa Benth inhibits the invasion and migration of MCF-7 breast cancer cells through the down regulation of matrix metalloproteinases. *Arab. J. Chem.* **6**, 49–58 (2013).

5. J. Cao, *et al.*, Sciadopitysin suppresses RANKL-mediated osteoclastogenesis and prevents bone loss in LPS-treated mice. *Int. Immunopharmacol.* **49**, 109–117 (2017).

6. J. Lan, *et al.*, Targeting FoxO proteins induces lytic reactivation of KSHV for treating herpesviral primary effusion lymphoma. *PLoS Pathog.* **19**, e1011581 (2023).

7. Y. Zhang, *et al.*, H3K27 acetylation activated-COL6A1 promotes osteosarcoma lung metastasis by repressing STAT1 and activating pulmonary cancer-associated fibroblasts. *Theranostics* **11**, 1473–1492 (2021).

8. Y. Cheng, *et al.*, Microfilaments and microtubules alternately coordinate the multi-step endosomal trafficking of Classical Swine Fever Virus. *J. Virol.* **95**, e02436-20, JVI.02436-20 (2021).

9. F. Chen, *et al.*, Histone deacetylase 3 aberration inhibits Klotho transcription and promotes renal fibrosis. *Cell Death Differ.* **28**, 1001–1012 (2021).

10. D. Huang, *et al.*, Crosstalk between PML and p53 in response to TGF-β1: A new mechanism of cardiac fibroblast activation. *Int. J. Biol. Sci.* **19**, 994–1006 (2023).

11. Y. Zhang, *et al.*, Novel roles of LSECtin in gastric cancer cell adhesion, migration, invasion, and lymphatic metastasis. *Cell Death Dis.* **13**, 593 (2022).

12. Y. Li, *et al.*, TLR9 agonist suppresses choroidal neovascularization by restricting endothelial cell motility via ERK/c-Jun pathway. *Microvasc. Res.* **141**, 104338 (2022).
